# Therapeutic efficacy of ramucirumab after lenvatinib for post-progression treatment of unresectable hepatocellular carcinoma

**DOI:** 10.1093/gastro/goaa042

**Published:** 2020-10-10

**Authors:** Atsushi Hiraoka, Takashi Kumada, Toshifumi Tada, Chikara Ogawa, Joji Tani, Shinya Fukunishi, Masanori Atsukawa, Masashi Hirooka, Kunihiko Tsuji, Toru Ishikawa, Koichi Takaguchi, Kazuya Kariyama, Ei Itobayashi, Kazuto Tajiri, Noritomo Shimada, Hiroshi Shibata, Hironori Ochi, Kazuhito Kawata, Hidenori Toyoda, Hideko Ohama, Kazuhiro Nouso, Akemi Tsutsui, Takuya Nagano, Norio Itokawa, Korenobu Hayama, Taeang Arai, Michitaka Imai, Yohei Koizumi, Shinichiro Nakamura, Kojiro Michitaka, Yoichi Hiasa, Masatoshi Kudo, the Real-life Practice Experts for HCC (RELPEC) Study Group and HCC 48 Group (hepatocellular-carcinoma experts from 48 clinics in Japan)

**Affiliations:** 1 Gastroenterology Center, Ehime Prefectural Central Hospital, Ehime, Japan; 2 Department of Gastroenterology and Hepatology, Ogaki Municipal Hospital, Gifu, Japan; 3 Department of Internal Medicine, Himeji Red Cross Hospital, Hyogo, Japan; 4 Department of Gastroenterology and Hepatology, Takamatsu Red Cross Hospital, Takamatsu, Japan; 5 Department of Gastroenterology and Hepatology, Kagawa university, Kagawa, Japan; 6 Department of Gastroenterology, Osaka Medical College, Osaka, Japan; 7 Division of Gastroenterology and Hepatology, Department of Internal Medicine, Nippon Medical School, Tokyo, Japan; 8 Department of Gastroenterology and Metabology, Ehime University Graduate School of Medicine, Ehime, Japan; 9 Center of Gastroenterology, Teine Keijinkai Hospital, Sapporo, Japan; 10 Department of Gastroenterology, Saiseikai Niigata Hospital, Niigata, Japan; 11 Department of Hepatology, Kagawa Prefectural Central Hospital, Takamatsu, Japan; 12 Department of Gastroenterology, Okayama City Hospital, Okayama, Japan; 13 Department of Gastroenterology, Asahi General Hospital, Asahi, Japan; 14 Department of Gastroenterology, Toyama University Hospital, Toyama, Japan; 15 Division of Gastroenterology and Hepatology, Otakanomori Hospital, Kashiwa, Japan; 16 Department of Gastroenterology, Tokushima Prefectural Central Hospital, Tokushima, Japan; 17 Hepato-biliary Center, Matsuyama Red Cross Hospital, Matsuyama, Japan; 18 Department of Hepatology, Hamamatsu University School of Medicine, Hamamatsu, Japan; 19 Department of Gastroenterology, Kindai University, Osaka, Japan

**Keywords:** hepatocellular carcinoma, ramucirumab, lenvatinib, sorafenib

## Abstract

**Background:**

Lenvatinib is used for unresectable hepatocellular carcinoma (u-HCC) as first-line, as well as second- and third-line therapy in Japan. We evaluated the therapeutic efficacy of newly developed ramucirumab when given after lenvatinib for post-progression treatment.

**Methods:**

Of 385 patients with u-HCC and treated with lenvatinib at 16 different institutions in Japan between May 2018 and January 2020, 28 who received ramucirumab as the next treatment were enrolled and therapeutic responses were evaluated in a retrospective manner.

**Results:**

The median age of the 28 patients given ramucirumab was 70 years and the median albumin-bilirubin score was −2.19. Of the 28 patients, 23 were male, 21 were classified as Child–Pugh A and 7 as Child–Pugh B, and 25 were Barcelona Clinic Liver Cancer Stage C. Ramucirumab was given as second-line therapy in 14, third-line in 9, and fourth-line in 5. Therapeutic response was obtained in only 26 patients; the objective response rate was 3.8% (1/26) and the disease-control rate was 42.3% (11/26), with a median period to progression of 2.0 months. The reasons for discontinuation of ramucirumab were progression of disease in 16 and Grade 3 adverse events (gastrointestinal bleeding, ascites) in 2.

**Conclusions:**

The anticipated therapeutic efficacy of ramucirumab for post-progression treatment following lenvatinib was not seen in our early experience.

## Introduction

Recently, several different systemic chemotherapy regimens that use a tyrosine kinase inhibitor (TKI) and/or molecular-targeting agent (MTA) for unresectable hepatocellular carcinoma (u-HCC) have become available. Sorafenib was developed as a first-line TKI [1, 2], after which regorafenib was introduced as a second-line drug in 2017 [3]. Nevertheless, a remaining important unmet need is the lack of therapeutic options for u-HCC patients who show sorafenib intolerance or regorafenib failure. Although lenvatinib was developed as a first-line TKI drug [4], it is given not only as a first-, but also as a second- and third-line treatment option in Japan [5–9].

In 2019, ramucirumab became available as a new second-line drug. Ramucirumab is a recombinant monoclonal human immunoglobulin IgG1 antibody-specific inhibitor of vascular endothelial growth factor 2 (VEGFR-2), which is an important primary driving factor for both physiological and pathological angiogenesis. Although no survival benefit was proved in the REACH trial, sub-analysis of patients with an alpha-fetoprotein (AFP) level ≥400 ng/mL revealed improved survival [10], thus the REACH-2 trial was planned and performed [11]. The REACH-2 trial demonstrated its clinical efficacy following sorafenib treatment as compared with a placebo [11]. However, lack of an established post-progression treatment option for patients with lenvatinib failure has become evident. In the present study, we aimed to elucidate the clinical features of post-progression systemic chemotherapy drugs, especially ramucirumab, for use following lenvatinib failure.

## Materials and methods

### Sources of patients

A total of 385 patients with u-HCC were treated with lenvatinib at specific institutions in Japan (Ehime Prefectural Hospital, Ogaki Municipal Hospital, Himeji Red Cross Hospital, Takamatsu Red Cross Hospital, Kagawa University, Osaka Medical School, Nippon Medical School, Ehime University Graduate School of Medicine, Teine Keijinkai Hospital, Saiseikai Niigata Hospital, Kagawa Prefectural Central Hospital, Okayama City Hospital, Asahi General Hospital, Toyama University Hospital, Otakanomori Hospital, Tokushima Prefectural Central Hospital, Matsuyama Red Cross Hospital, and Hamamatsu University School of Medicine) between May 2018 and January 2020. Among them, 28 who received ramucirumab as post-progression treatment and showed therapeutic response were evaluated in a retrospective manner.

### Basal hepatic disease related to HCC

HCC due to hepatitis C virus (HCV) was judged when the anti-HCV was positive, whereas HCC due to hepatitis B virus (HBV) was judged when the hepatitis B virus surface antigen (HBsAg) was positive.

### Methods for hepatic reserve function and therapeutic-response assessments

Child–Pugh classification [12] and albumin-bilirubin (ALBI) grade were used for assessment of the hepatic reserve function. The ALBI grade was calculated with serum-albumin and total-bilirubin values using the following formula: ALBI score = [log10 bilirubin (µmol/L) × 0.66] + [Albumin (g/L) × −0.085)], with the result defined by the following scores: ≤−2.60, Grade 1; >−2.60 to ≤−1.39, Grade 2; and >−1.39, Grade 3 [13–15]. To perform more detailed evaluations of patients with the middle ALBI grade of 2, we used a revised grading system consisting of four levels that included sub-grading for the middle grade of 2 (2a and 2b) based on an ALBI score of −2.27 as the cut-off, which was previously developed based on the value for indocyanine green retention after 15 min (ICG-R15) of 30% [16, 17].

Local physicians at each participating hospital independently evaluated HCC status using dynamic computed tomography (dy-CT) and/or magnetic resonance imaging (MRI) procedures performed at 4 or 8 weeks after the introduction of post-progression treatment (sorafenib or ramucirumab) based on the Response Evaluation Criteria in Solid Tumors (RECIST) guidelines version 1.1 [18], when possible.

### HCC diagnosis and treatment

Based on an increasing course of AFP, as well as dy-CT [19], MRI [20, 21], contrast-enhanced ultrasonography with perflubutane (Sonazoid^®^, Daiichi Sankyo Co., Ltd, Tokyo, Japan) [22, 23], and/or pathological findings, HCC was diagnosed. We used Barcelona Clinic Liver Cancer (BCLC) stage [24] and tumor node metastasis (TNM) stage, determined as previously reported in a study for the TNM staging of HCC conducted by the Liver Cancer Study Group of Japan (LCSGJ) 6th edition [25] (TNM-LCSGJ), to evaluate tumor progression. Written informed consent for MTA treatment was obtained from all of the patients. The protocol used in the present study was approved by the Institutional Ethics Committee of Ehime Prefectural Central Hospital (IRB No. 30-66).

### Ramucirumab treatment and assessment of adverse events

Ramucirumab was given as an intravenous injection at a dose of 8 mg/kg once every 2 weeks. According to the guidelines for the administration of ramucirumab, the dose should be reduced or the treatment should be interrupted when a patient develops any Grade 3 or more severe adverse event (AE) or if any unacceptable Grade 2 drug-related AE occurs. AEs were assessed according to the National Cancer Institute Common Terminology Criteria for Adverse Events, version 4.0 [26]. The worst grade for each AE during the present observation period was recorded. If a drug-related AE occurred, dose reduction or temporary interruption was maintained until the symptom was resolved to Grade 1 or 2, according to the guidelines provided by the manufacturer.

### Statistical analysis

Statistical analyses were performed using the Kaplan–Meier method or a log-rank test. A *P*-value <0.05 was considered statistically significant. Easy R (EZR), version 1.29 (Saitama Medical Center, Jichi Medical University, Saitama, Japan) [27], a graphical user interface for R (The R Foundation for Statistical Computing, Vienna, Austria), was used for all statistical analyses.

## Results

### Patient characteristics

Among the 385 patients treated with lenvatinib during the study period, 28 who subsequently received ramucirumab as post-progression treatment were analysed. Their median age was 70 years and 23 were male. Six were diagnosed pathologically and the others were using the diagnostic algorithm for HCC [24]. No patient had any known past history of chronic hepatic failure, chronic respiratory diseases, or chronic renal failure. Twenty-one were classified as Child–Pugh A and seven as Child–Pugh B, and the median ALBI score was −2.19. BCLC stage C was noted in 25. All had undergone lenvatinib treatment, with ramucirumab used as second-line treatment in 14, as third-line in 9, and as fourth-line in 5 (Table 1).

**Table 1:** Clinical features of 28 unresectable hepatocellular-carcinoma patients treated with ramucirumab

Factor	Value
Age, years^a^	70 (60–76)
Gender, male:female	23:5
Etiology, HCV:HBV:alcohol:others	8:7:4:9
BMI, kg/m^2^^a^	23.2 (21.1–24.3)
EOCG PS, 0:1	18:10
Platelets, ×10^4^/µL^a^	13.9 (11.2–19.2)
AST, IU/L^a^	51 (36–68)
ALT, IU/L^a^	32 (20–57)
Total bilirubin, mg/dL^a^	0.9 (0.7–1.2)
Albumin, g/dL^a^	3.5 (3.3–3.7)
Prothrombin time, %^a^	91 (82–97)
NH3, μg/dL^a^	50 (36–60)
Child–Pugh score, 5:6:7:8:9	9:12:6:0:1
Modified ALBI, 1:2a:2b	2:9:17
ALBI score^a^	−2.19 (−2.36 to −1.96)
Positive for MVI (*n* = 10)^b^, Vp2:Vp4:Vv2	7:3:2
Positive for EHM (*n* = 18)^b^, lung:bone:LN:others	13:5:5:2
TNM stage of LCSGJ 6th, III:IVa:IVb	4:6:18
BCLC stage, B:C	3:25
AFP, ng/mL	3,019 (989–8,189)
History of MTAs^b^, LEN:SOR:REG	28:14:5
Previous treatment, LEN:SOR:TACE	22:5:1
Use of ramucirumab, 2nd:3rd:4th-line	14:9:5

a Median (interquartile range). b There are duplicate cases; HCV, hepatitis C virus; HBV, hepatitis B virus; others, patients without viral infection or alcohol abuse; BMI, body mass index; ECOG PS, Eastern Cooperative Oncology Group performance status; AST, aspartate transaminase; ALT, alanine aminotransferase; ALBI, albumin-bilirubin; MVI, main vessel invasion; Vp2, tumor invasion to secondary portal branch; Vp4, tumor invasion to main portal branch; Vv2, tumor invasion to hepatic vein trunk; EHM, extra-hepatic metastasis; LN, lymph node; TNM-LCSGJ 6th, tumor node metastasis stage by Liver Cancer Study Group of Japan, 6th edition; BCLC, Barcelona Clinic Liver Cancer stage; AFP, alpha-fetoprotein; MTA, molecular-targeting agent; LEN, lenvatinib; SOR, sorafenib; REG, regorafenib; TACE, transcatheter arterial chemo-embolization.

### Therapeutic efficacy

**Figure 1.**
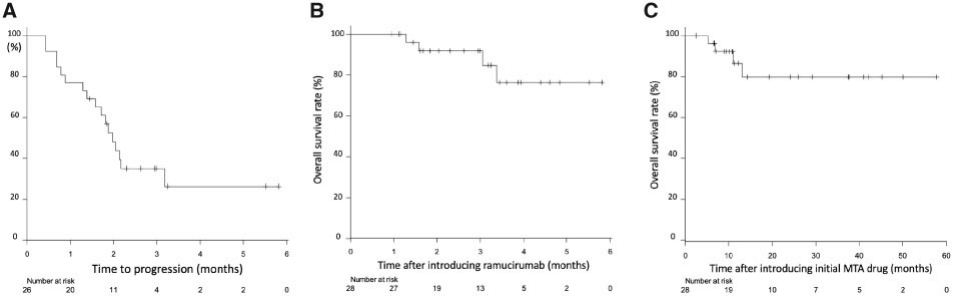
Overall survival of 28 patients with unresectable hepatocellular carcinoma treated with ramucirumab. (A) Time to progression after starting ramucirumab treatment. (B) Overall survival (OS) after introducing ramucirumab. (C) OS after introducing initial molecular-targeting agent.

Among these 28 patients, therapeutic response according to RECIST version 1.1 was obtained in 26. Partial response (PR) was observed in only 1 and stable disease (SD) was noted in 10, whereas progressive disease (PD) was seen in 15, with an objective response rate (ORR) of 3.8% (1/26) and a disease-control rate (DCR) of 42.3% (11/26) (Table 2). Until the end of January 2020, ramucirumab treatment was stopped in 18; the major reasons for discontinuation were PD in 16 and AE (Grade 3) in 2 (gastrointestinal bleeding, ascites). The median time to progression (TTP) was 2 months [95% confidential interval (CI), 1.4–3.2 months] (Figure 1A). There were no significant differences for TTP and DCR between patients who only received lenvatinib and those treated with multiple MTA drugs, including lenvatinib before ramucirumab (1.8 vs 2.1 months, *P* = 0.789) (38.5% vs 46.1%, *P* = 1.0). TTP was also not significantly different between Child–Pugh class A and B classification (2.0 vs 2.1 months, *P* = 0.986). At the end of the study period, more than half of the patients had survived, thus the median survival time after introducing ramucirumab and the initial MTA drug were not reached (Figure 1B and C).

**Table 2.** Therapeutic results in 28 unresectable hepatocellular-carcinoma patients treated with ramucirumab

Best therapeutic response (RECIST)^a^, CR: PR: SD: PD: NE	0:1:10:15:2
Reason for RAM discontinuation (*n *=* *18), PD: AE	16:2
Observation period after starting initial MTA drug (IQR), days	358 (281–952)
Observation period after starting RAM (IQR), days	90 (50–112)
Time to progression (IQR), days	56 (37–72)

a Evaluated according to Response Evaluation Criteria in Solid Tumors (RECIST). CR, complete response; PR, partial response; SD, stable disease; PD, progression disease; NE, not examined; RAM, ramucirumab; AE, adverse event; MTA, molecular-targeting agent; IQR, interquartile range.

### Adverse events

Grade 3 AEs noted in the 28 patients were hepatic coma (*n* = 1; 3.6%), gastrointestinal bleeding (duodenal ulcer: *n* = 1; 3.6%), ascites (*n* = 1; 3.6%), fatigue and appetite loss (*n* = 1; 3.6%), and fever (*n* = 1; 3.6%), whereas Grade 1/2 AEs included ascites or pretibial edema (*n* = 6; 21.4%), fatigue and appetite loss (*n* = 5; 17.8%), diarrhea (*n* = 3; 10.7%), and fever (*n* = 1; 3.6%) (Table 3).

**Table 3.** Adverse events seen in 28 unresectable hepatocellular-carcinoma patients treated with ramucirumab

Adverse event	Grade 1 or 2	Grade 3
Ascites, pretibial edema	6	1 (ascites)
Fatigue and appetite loss	5	1
Diarrhea	3	0
Fever	1	1
Hepatic coma	0	1
Gastrointestinal bleeding	0	1 (duodenal ulcer)

**Table 4.** Inhibitory effects of molecular-targeting agent on target kinase

Kinase	Sorafenib	Regorafenib	Lenvatinib	Ramucirumab
	IC_50_ (nM)	IC_50_ (nM)	IC_50_ (nM)	IC_50_ (nM)
VEGFR1		13 ± 0.4	4.7	
VEGFR2	15	4.2 ± 1.6	3.0	0.8
VEGFR3	20	46 ± 10	2.3	
RET		1.5 ± 0.7	6.4	
FGFR1	580	202 ± 18	61	
FGFR2			27	
FGFR3			52	
FGFR4			43	
KIT		7 ± 2	85	
PDGFR1			29	
PDGFRb	57	22 ± 3	160	
RAF1		2.5 ± 0.6	1,600	
c-Raf	6			
B-Raf V600E	38	19 ± 6		
WT B-raf	22	28 ± 10		
FLT3	58			
TIE2		311 ± 46		

Obtained from interview form for each drug. IC_50_, inhibitory concentration 50%; VEGFR, vascular endothelial growth factor receptor; RET, rearranged during transfection; FGFR, fibroblast growth factor receptor; PDGFR, platelet-derived growth factor receptor; RAF, Rapidly Accelerated Fibrosarcoma; FLT3, FMS-like tyrosine kinase 3; TIE, tyrosine kinase with Ig and EGF homology domains.

## Discussion

In the present analysis, the ORR and DCR for ramucirumab as post-progression treatment following lenvatinib were 3.8% and 42.3%, respectively. In previous reports [5–9], the therapeutic efficacy of lenvatinib did not show a significant difference between u-HCC patients with or without a past history of MTA treatment. A similar result was observed in the present study. There were no significant differences for either TTP or DCR between patients who received only lenvatinib and those were treated with multiple MTA drugs, including lenvatinib, prior to ramucirumab.

Few reports regarding the therapeutic efficacy of ramucirumab given following lenvatinib for post-progression have been presented. In the REACH-2 trial, the ORR and DCR for ramucirumab given as a second-line treatment after sorafenib [11] were superior as compared with those in the present study. Recently, Kuzuya *et al.* [28] reported a high DCR (80%) with ramucirumab in 10 patients with unresectable HCC, of whom 8 (80%) were treated with ramucirumab as second-line treatment following lenvatinib. In contrast, ramucirumab was used as a third- or fourth-line treatment more frequently (50%) in the present study than in that report. Interestingly, in the present cohort, there was no significant difference for DCR between patients who received ramucirumab as second-line and those who received it as third-/fourth-line treatment. Our results suggest that the therapeutic potential of ramucirumab given as post-progression treatment after lenvatinib might be not sufficient.

Abnormal expression of FGF19-FGFR4 has been reported as an oncogenic-driver pathway for HCC [29]. Among the four MTA drugs available in Japan at the time of writing (early 2020), only lenvatinib is known to inhibit FGFR 4 (Table 4). In another study, sorafenib administration did not increase the level of FGF19 from the baseline, whereas the level of FGF19 was significantly increased compared with the baseline in patients given lenvatinib (*P* < 0.001), and the FGF19 level was higher in the patients given lenvatinib than in those given sorafenib at any point during the study period (*P* < 0.05) [30]. FGFR signals via the autocrine loop of FGFR4 corresponding to FGF19 have been reported [31–34] and an elevated level of FGF19 in serum is thought to be a result of the suppression of FGFR4 expression by lenvatinib. When therapeutic failure is observed in patients receiving lenvatinib, FGFR4 expression is presumed to exist, thus HCC may have acquired resistance to VEGF signal suppression at the time of lenvatinib failure. As a result, it is expected that not only ramucirumab, but also other MTAs (sorafenib and regorafenib), might have a lower therapeutic effect than expected in patients with lenvatinib failure. Although lenvatinib was developed as a first-line agent, it has also been used for late-line treatments in Japan [5–9]. In contrast, sorafenib has not been shown to have potential for post-progression treatment following lenvatinib. In our Child–Pugh A patients, the ORR and DCR for sorafenib given for PD in patients who had received lenvatinib were 0.0% and 16.7%, respectively, whereas those for ramucirumab were 0.0% and 36.8%, respectively (data not shown). Thus, a new unmet clinical need is now apparent, as there is no established post-progression therapy for patients with lenvatinib failure.

This study has some limitations, including its retrospective nature. In addition, the number of cases analysed was too few to obtain concrete conclusions. We did not examine FGF-19-FGFR4 expression in the present cohort, thus further investigations and accumulation of more records of patients given ramucirumab as post-progression treatment following lenvatinib failure are needed. Nevertheless, our findings indicate that the therapeutic efficacy of ramucirumab after failure with lenvatinib might be limited.

In our early experience, the anticipated therapeutic efficacy of ramucirumab given following lenvatinib treatment has not been observed. It is difficult to fully explain our results at this time; determination of the best strategic order of administration of these drugs is an important issue to obtain prognosis improvement in affected patients.

### Statement of ethics

The present study protocol was approved by the Institutional Ethics Committee of Ehime Prefectural Central Hospital [IRB No. 30-66]. The research was conducted ethically in accordance with the World Medical Association Declaration of Helsinki.

## Authors’ contributions

A.H. and T.K. conceived of the study and participated in its design and coordination. A.H., Ku.T., Ko.T., E.I., K.Kar., H.Oc., Ka.T., M.H., T.A., N.S., T.I., A.T., H.S., T.T., K.N., N.I., K.J., Y.H., K.M., M.I., M.A., K.H., T.N., Y.K., S.F., H.Oh., K.Kaw., S.N., J.T., C.O., and M.K. performed data curation. A.H. performed statistical analyses and interpretation. A.H. and T.K. drafted the text. All authors have read and approved the final version of the manuscript.

## Funding

None to declare.
